# Fractalkine in Health and Disease

**DOI:** 10.3390/ijms25158007

**Published:** 2024-07-23

**Authors:** Claudia Rodriguez, Luisa Chocarro, Miriam Echaide, Karina Ausin, David Escors, Grazyna Kochan

**Affiliations:** Oncoimmunology Unit, Instituto de Investigación Sanitaria de Navarra (IdiSNA), Navarrabiomed-Fundación Miguel Servet, Universidad Pública de Navarra (UPNA), Hospital Universitario de Navarra (HUN), 31008 Pamplona, Spain; claudia.rodriguez.neira@navarra.es (C.R.); luisa.chocarro.deerauso@navarra.es (L.C.); miriam.echaide.gorriz@navarra.es (M.E.); karina.ausin.perez@navarra.es (K.A.)

**Keywords:** Neurotactin, immunotherapy, T-cell, dendritic cell, immune checkpoint, PD-1, PD-L1, FKN

## Abstract

CX3CL1 is one of the 50 up-to-date identified and characterized chemokines. While other chemokines are produced as small, secreted proteins, CX3CL1 (fractalkine) is synthetized as a transmembrane protein which also leads to a soluble form produced as a result of proteolytic cleavage. The membrane-bound protein and the soluble forms exhibit different biological functions. While the role of the fractalkine/CX3CR1 signaling axis was described in the nervous system and was also related to the migration of leukocytes to sites of inflammation, its actions are controversial in cancer progression and anti-tumor immunity. In the present review, we first describe the known biology of fractalkine concerning its action through its cognate receptor, but also its role in the activation of different integrins. The second part of this review is dedicated to its role in cancer where we discuss its role in anti-cancer or procarcinogenic activities.

## 1. The Chemokine CX3CL1, Fractalkine

Fractalkine (CX3CL1, FKN) is a transmembrane chemokine and the only member of the CXXXC subfamily. The gene coding for human CX3CL1 is located on chromosome 16q13. The full-length gene spans 12,590 base pairs and contains three exons [1]. Fractalkine is a type I transmembrane protein composed of a 373 amino acid polypeptide chain organized into four structural domains: an amino-terminal chemokine domain of 76 residues followed by a 241-aa mucin-like stalk connecting the N-terminal chemokine domain with a 19-aa transmembrane α helix. The intracellular cytoplasmic tail is short, comprising 37 residues [2].

Fractalkine was first identified by Pan and co-workers in the brain and was named neurotactin [3]. In parallel, it was identified by Bazan and colleagues who characterized the expression of this chemokine in different tissues [4]. Fractalkine protein was found to be highly expressed in the brain, lung, and heart. It is also expressed at lower levels in other organs such as kidney, pancreas, skeletal muscles, colon, and testis. Fractalkine expression was found in several cell types, including neurons [5], glial cells [6], endothelial cells [7], airway smooth muscles cells [8], fibroblasts, bronchial epithelial cells [9], and keranocytes [10]. Fractalkine expression is upregulated by classical proinflammatory cytokines such as TNFα, IL1β, and IFNγ. Indeed, FKN can be also be upregulated via autocrine secretion of cytokines [11]. FKN expression is transcriptionally upregulated through binding of NF-κB, STAT-1, and STAT-2 to its promoter [12]. 

**Figure 1:**
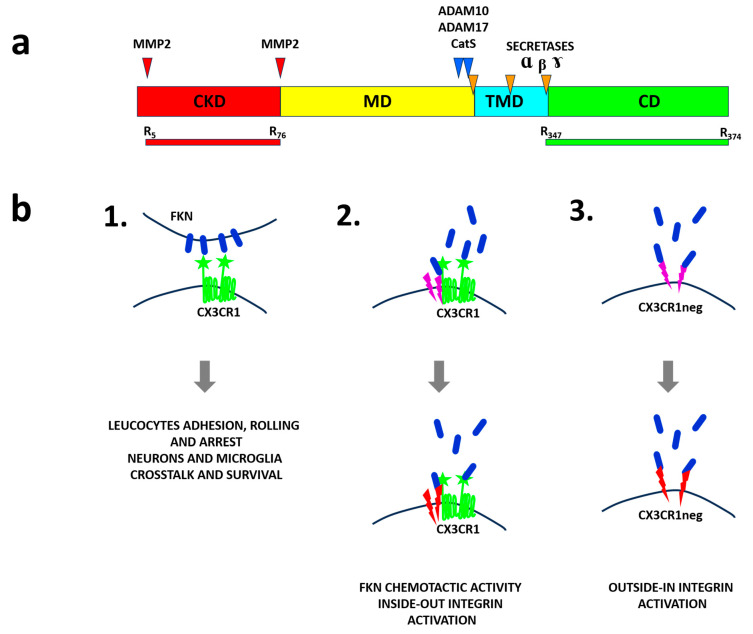
FKN polypeptides. Membrane-bound fractalkine can be shed by different proteases resulting in soluble forms. (**a**) Arrows show sites of proteolytic digestion and the proteases responsible for it. ADAM10, ADAM17, and CatS (blue); MMP2 (red); and secretases α, β, and γ (orange). Below the FKN full-length (FL) molecule, the collection of polypeptides generated via proteolytic cleavages are shown. The first and last amino acid residues are marked. (**b**) Function of FKN. The membrane-bound form (in blue) causes adhesion of leucocytes to the endothelial surface. It is also responsible for neuron–microglia crosstalk and survival (1). The soluble form (in blue) acts as chemoattractant for different cells which express its receptor CX3CR1 on their surface. Interaction with its receptor results in a signaling cascade (among others, integrin activation and inside-out activation mechanism, shown as colored serrated arrows). (2) Soluble fractalkine in the absence of its receptor CX3CR1 can bind to integrins in their inactive form (binding to the alternative site 2) and activate them (outside-in activation, shown as colored serrated arrows) that is followed by binding to their corresponding ligands (3).

Fractalkine is synthetized as a membrane-bound form that serves as an endothelial cell adhesion and migration molecule for a variety of immune cell types including natural killer (NK) cells, CD3 T cells, monocytes, and dendritic cells (DCs) (Figure 1). These immune cell types can upregulate its receptor CX3CR1 on their surface, facilitating their migration to distant sites expressing fractalkine [2]. Fong and colleagues showed that the highly glycosylated mucin domain is responsible for extending the chemokine domain from endothelial cells towards flowing leucocytes [13]. Moreover, the glycosylation of the mucin domain is responsible for protein stability [14]. Additionally, the N-terminal glutamine in fractalkine undergoes modification to pyroglutamate. This modification, which affects protein stability and interactions with its receptor has been found in other chemokines [15]. Indeed, Kehlen and co-workers demonstrated that modification of the N-terminal glutamine to pyroglutamate is necessary for the full biological activity of FKN [15]. In agreement with this, it has been described that this type of modification also improves protein stability [16].

FKN can oligomerize and this has been shown to favor interaction with its receptor leading to strengthened adhesion between cells [17,18]. Membrane-bound FKN (mCX3CL1) can be recycled to early/recycling endosomes where it colocalizes with syntaxin-13 and VAMP-1 [19]. The authors of this study suggested that part of FKN processing in endosomes is mediated by TACE (ADAM17). It was also hypothesized that intracellular storage of FKN can be the protein source for acute upregulation of mCX3CL1. Fan and colleagues demonstrated that FKN is a substrate for α, β, and γ secretases as well, and that its processing was involved in the generation of amyloid-β fragments from the amyloid protein precursor [20]. Interestingly, these authors found that the intracellular domain of FKN released via intracellular processing can translocate to the cell nucleus altering gene expression profiles. These results demonstrated the existence of a back signaling function for FKN. 

CX3CR1 was identified as a unique specific cellular receptor for fractalkine, although the FKN chemokine domain (cdFKN) can also bind to integrins αvβ3, α4β1 α5β1, and αIIb β3. A mutational study of the chemokine domain showed that mutating Lys36 and Arg37 completely abrogated integrin activation capacity [21]. In contrast, mutation of Arg47 completely abolished the chemotaxis-promoting activity of soluble FKN, as well as intracellular Ca^2+^ mobilization in CX3CR1-positive cells. Mutation of Lys31 only slightly affected chemotaxis-promoting activity while the equivalent mutation in IL8 reduced affinity to its receptor [22]. 

## 2. Soluble Fractalkine 

**Figure 2:**
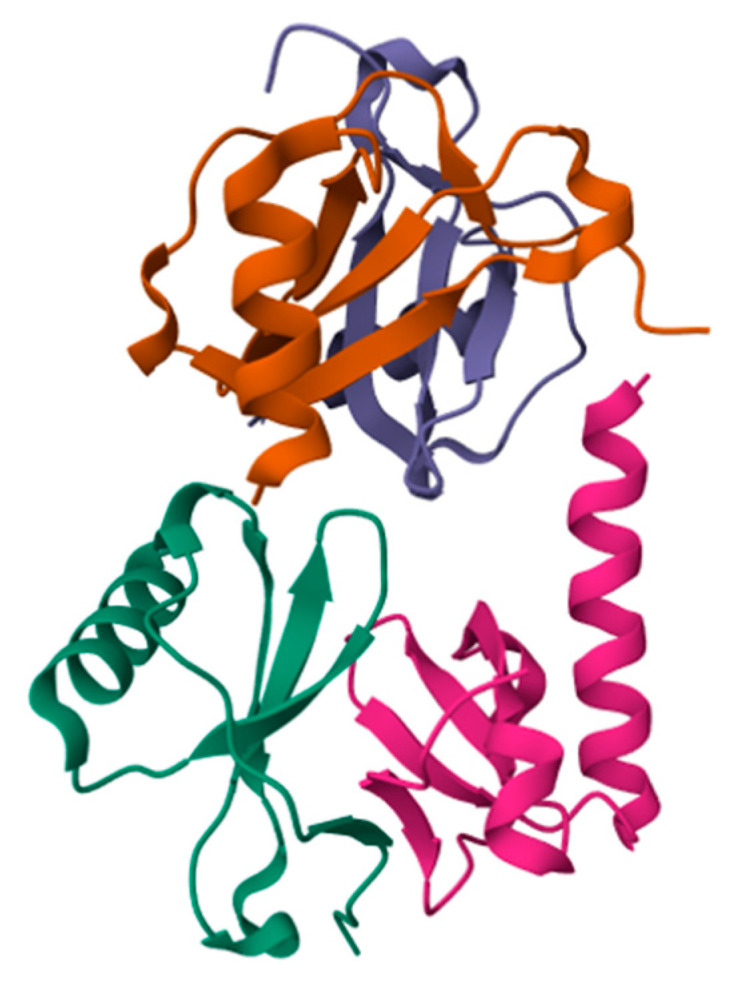
PDB structure of chemokine domain of FKN (PDB 1f2l). The chemokine domain of FKN in the crystal showed a quaternary arrangement [28]. In the figure, four FKN molecules are visible and are indicated in different colors. Nevertheless, FKN does not form oligomers in solution. However, it cannot be disregarded that, in the presence of other molecules, it may oligomerize.

Fractalkine is susceptible to undergo proteolytic cleavage by metalloproteases such as ADAM-10, ADAM-17 (TACE) and MMP-2 [23,24,25] (Figure 1). Hence, a soluble version of FKN can be released into the extracellular space as 80–85 kDa protein, and acts as a chemoattractant in a similar way to other chemokines (Figure 2). During this proteolytic processing, the mucin stalk is cut close to the transmembrane domain, releasing the soluble form. Originally, it was suggested that dibasic amino acids (R326-R327) at the N-terminus of the transmembrane domain constitute a potential protease cleavage site [4]. However, Garton and colleagues [23] suggested after performing a mutational deletion study that FKN cleavage is determined by the structure of the juxtamembrane region rather than the presence of specific amino acid sequences. FKN can also be processed by other proteases, for example cathepsin S, leading to the release of a soluble FKN protein in vascular smooth muscle cells [11] (Figure 1a). Cathepsin S expression is stimulated by proinflammatory factors such as IL1β, TNFα, and IFNγ. When FKN is processed by cathepsin S, the soluble product presents a mass of about 55 kDa. It has been proposed that proteolytic processing leading to the release of soluble FKN takes place within recycling endosomes. Moreover, Dean and colleagues in the proteomics analysis of matrix metalloprotease 2 substrate degradome identified MMP2 as a protease that can generate soluble FKN lacking its mucin domain [26]. Moreover, they have shown that MMP2 can generate soluble FKN lacking the first four amino acids. Inoue and collaborators showed that this shortened form of FKN (5–78 aa) acts as an antagonist of FKN in a murine lupus nephritis model [27].

It is likely that different forms of soluble FKN may exhibit differential activities (Figure 1b). Hence, the affinity of soluble FKN versions to its receptor was evaluated by Finneran and colleagues [29]. In the study, sFKN (1–337 aa) and a shorter version of the FKN chemokine domain without the mucin-like domain (cdFKN 1–78 aa) were assessed for binding activities (Figure 2). While they observed only a modest difference in affinity to its receptor in competition and functional assays, the authors showed differences in the potency of β-arrestin recruitment. The authors observed that both forms of FKN were able to cause TNFα and IL1β downmodulation. However, sFKN was also more effective in modulating TNFα secretion. Surprisingly, while low concentrations of sFKN decreased TNFα secretion, higher sFKN concentrations had the opposite effect. Interestingly, similar results were observed for IL6 secretion. The cdFKN did not have such profound effects on modulation of cytokine expression when compared to sFKN. Moreover, the authors did not observe receptor surface internalization after incubation with high concentrations (100 nM) of sFKN and only a minor change (14%) after incubation with cdFKN. Use of antagonists of integrins interacting with FKN in CX3CR1^−^ lines have shown no difference in the TNFα secretion profile between control (untreated) and antagonist-treated cells [29]. Differences in β-arrestin activation were consistent with calcium influx. The sFKN-containing mucin domain was much stronger as a calcium influx inducer. This pointed to a role of the mucin stalk in binding and activation of the FKN receptor. These data were contradictory to a study by Nakayama and co-workers, who observed downmodulation of receptors after interaction with cdFKN [30].

## 3. Fractalkine Receptor and Signal Transduction

The specific receptor for FKN, CX3CR1, was identified by Combadiere and collaborators [31] and in parallel by Imai and collaborators [32]. These authors showed an active calcium flux response in cells overexpressing CX3CR1 after stimulation with cdFKN (1–76 aa). 

CX3CR1 is expressed on different immune cells, including NK cells, T cells, monocytes, dendritic cells, and microglia in the brain [2,5,32,33,34,35,36,37]. CX3CR1 was previously known as CMKBRL1/V28, and, interestingly, it serves as a co-receptor for certain isolates of HIV-2 and HIV-1. CX3CR1 belongs to the GPCR family and also serves as a receptor for Eotaxin-3 (CCL26) [30]. While both FKN and Eotaxin-3 chemokines induce calcium flux in cells expressing the receptor in a pertussis toxin (PTX)-dependent manner, receptor desensitization was 10-fold more potent for CX3CL1 than for Eotaxin-3. Moreover, only the interaction of CX3CR1 with FKN resulted in receptor internalization in a dose-dependent manner [30]. It is worth noting that these two chemokines are induced by different cytokine profiles. For example, FKN expression is induced by proinflammatory cytokines, while Eotaxin-6 is induced by anti-inflammatory cytokines. So far, some genetic variants of the receptor have been identified as linked to some pathologies. The CX3CR1 T280M variant is associated with a lower risk for coronary disease, while the V249I variant presents the opposite effect on the prevalence of coronary disease [34]. The CX3CR1 V249I variant is also associated with obesity [38]. Interestingly, both genetic variants have been associated with rapid progression of HIV in the homozygous haplotype in the Caucasian population [39].

CX3CR1 is also known as the G-protein-coupled receptor 13 (GPR13) belonging to the G-protein-coupled receptor 1 (GPCR1) family. Binding of CX3CR1 to FKN induces a conformational change and dissociation of the α, β, and γ subunits of the heterotrimeric G complex [40]. The separated G protein components Gα and Gβγ then activate further downstream signaling pathways including the PLC/PKC, PI3K/AKT/NFκB, Ras/Raf/MAPK, and CREB pathways. The activities of these signaling pathways are fairly well known and explain the functions exerted by FKN, including regulation of proliferation, cell migration, and resistance to apoptosis, among others. Intracellular calcium release has been described in breast cancer cells [41] and in rat brain microglia [42], regulating cell proliferation and migration. The PI3K and MAPK pathways are activated by CX3CR1 in endothelial cells, contributing to their proliferation and angiogenesis [43] in clear-cell renal carcinoma and prostate cancer cells involved in metastasis and migration [44,45]. Other pathways such as the Src-FAK and JAK2-STAT3 pathways, have also been described in several models, regulating cell survival, proliferation, and migration [46,47,48].

## 4. Integrins 

Integrins are well-known molecules present on the cell surface, with critical roles in cell adhesion and migration. Fujita and colleagues found that the chemokine domain of FKN (Figure 2) directly binds to integrins αvβ3 and α4β1 at their classical binding site (site 1) [21] (Figure 1b). They carried out a mutational study and showed that mutations of K36E/R37E affected signal transduction through integrin activation even though FKN binding to CX3CR1 was not affected. The authors uncovered that FKN-mediated chemotaxis is a process dependent on CX3CR1 binding but not on integrin binding. Therefore, a model was proposed in which FKN binding to CX3CR1 causes the recruitment of integrins, leading to signal transduction that induces a conformational change in integrins followed by the formation of a ternary complex that promotes leukocyte migration through the endothelial layer. In this study, it was shown that mutations in K36R37 affected leukocyte migration in a peritonitis model. In a subsequent study, the same authors demonstrated that cdFKN activates integrins in a cell-free assay. Moreover, FKN enhanced integrin binding to their cognate ligands on the cell surface. FKN bound to integrins in the absence of CXCR1 in their inactive form. In this inactive conformation, the RGD-binding site of integrins is hidden. Hence, in this process, FKN bound to the alternative site, named “site 2” (Figure 1b). The authors of the study demonstrated that this process is cell-type-independent and did not require an inside-out signaling process. Analysis of FKN binding to site 2 indicated that FKN additionally binds and activates integrins α4β1 and α5β1. In contrast, the KFN K36/R37 mutant could not activate these integrins [49]. A recent study performed by Takada and colleagues showed that FKN can also bind to integrin αIIbβ3 present on platelets. The authors characterized the binding of chemokines to soluble αIIbβ3 as well as the binding to integrin proteins exposed on the cell surface. Cell surface-bound integrins were activated more effectively and required significantly lower chemokine concentrations. These key findings indicated that integrin αIIbβ3 can be activated independently from the canonical inside-out signaling pathway (Figure 1b). The authors concluded that this activation of αIIbβ3 can be a possible link between inflammation and thrombosis [50]. 

## 5. Biological Functions of FKN 

Fractalkine is expressed by a variety of cell types and tissues, playing an important role in homeostasis. More specifically, studies in knock-out mice showed that FKN is mainly expressed in non-pathological conditions in neurons and epithelial cells from the lung, kidney, and intestine [51,52]. The membrane-bound FKN can be expressed following stimulation with proinflammatory cytokines [4] on the surface of endothelial cells [53] and smooth muscle cells [54]. The first functions of FKN in homeostasis were associated to its neuroprotective effects during brain injury, and the evidence shows that FKN expression by other cell types is related to protection from apoptosis [2,55]. Indeed, it has been shown that FKN expression is induced in a p53-dependent manner following genotoxic damage caused by irradiation [56]. FKN also acts as an adhesion molecule in endothelial cells interacting with its receptor CX3CR1 which is expressed by leucocytes. In this way, FKN mediates the capture, arrest, and activation of leucocytes on the endothelial surface in conditions of physiologic flow [33]. The capacities of FKN to mediate cell adhesion have been evaluated in adhesion assays of the macrophage cell line THP-1 to immobilized FKN, compared to adhesion mediated by an intracellular adhesion molecule (ICAM-1) or fibronectin (FN). Interestingly, a more efficacious cell adhesion was achieved by FKN compared to ICAM-1 or FN. Then, the adhesion capacities of these ligands were tested in combination, which demonstrated that the combination of these ligands with FKN resulted in much stronger adhesion. Use of soluble FKN or specific antibodies towards integrins inhibited cell adhesion to baseline, confirming that all factors contributed cooperatively to effective binding [57]. 

The interaction between FKN and its receptor is independent of G protein [32], moreover FKN activates G proteins to enhance integrin avidity binding [58]. Interactions dependent on G proteins induced shedding signals for metalloproteases to generate soluble FKN. Interestingly, sFKN acts as a chemoattractant and causes migration of leucocytes to sites following an increasing chemokine gradient. Soluble FKN also interacts with its receptor, inducing an increase in intracellular Ca^2+^ in the cytoplasm and β-arrestin recruitment followed by internalization of the receptor. 

Fujita and colleagues demonstrated that both soluble and membrane forms of FKN can bind integrins. These authors proved that sFKN supports cell adhesion through binding to αvβ3 in the K562 cell line, which lacks CX3CR1 expression [21]. In an elegant continuation of this study, the authors identified different integrins as FKN binding partners. Moreover, they characterized the molecular mechanisms of their interactions and functions [21,49,50].

There is an unquestionable role for FKN in the central nervous system where the CX3CL1–CX3CR1 axis is an important factor for the development of the nervous system and neurogenesis in the mammalian brain throughout adult life [59]. The FKN–CX3CR1 signaling axis is key for brain homeostasis, neuronal plasticity, and transmission of synaptic signals. It is important to highlight that the peptidic fragment of the FKN cytoplasmatic domain generated after FKN processing and shedding by α, β, and γ secretases acts as a transcriptional activator for a large number of genes. These genes play a critical role for cell growth and differentiation, including TGFβ3 and regulators of SMAD signaling pathways among others. The authors of the study demonstrated the overturning of neuronal loss and the reduction in amyloid deposition in a murine Alzheimer disease model mediated by the transcriptional activity of the FKN cytoplasmic domain fragment [20]. Transcriptional activities for FKN fragments have also been demonstrated in other studies. For example, a soluble protein fragment (1–336) from mouse CX3CL1 increased the transcriptional upregulation of mRNAs encoding CD5L, LDLRAD3, MUC16, CTLA2A, FCRL1, CD200R4, FOXP2, N4BP3, and CST7 while decreased those encoding AIPL1, PDC, IQCN, CHRNB4, RHPN2, and ANKRD33 in microglia from the retina in a rd10 mouse model for retinitis pigmentosa [60]. Another example is found in the transcriptional downmodulation of SAA2 mRNA by human CX3CL1, CXCL12, and CXCL16 in human LN229 cells which is otherwise elevated by temozolomide treatment [61]. In mouse neurons, transgenic mutant FKN protein (deletion p.X28_X271del) containing a C-terminal domain and a signal peptide increased the expression of mouse CDCA7, ZPLD1, NBL1, BRINP2, SPON2, CPNE6, ASPA, FRMD7, SDK2, NDNF, SEMA5A, and APCDD1 mRNA in the mouse hippocampus [20]. Indeed, binding of human CX3CL1 gene with ZNF606, IRX5, and IRX6 proteins has been demonstrated through yeast one-hybrid cloning [62]. Interestingly, human AZU1 has been shown to be involved in the biosynthesis of CX3CL1 protein [63].

FKN is also involved in a wide range of physiological processes. For example, a study by Lee and Olefsky demonstrated that the FKN–CX3CR1 axis regulates pancreatic β cell function and insulin secretion. The authors demonstrated that FKN was necessary for insulin secretion as well as glucose tolerance in a murine model. Diminished insulin secretion and glucose intolerance was observed following the administration of anti-FKN antibody. Indeed, FKN administration in vivo increased glucose tolerance and insulin secretion. These therapeutic effects were not observed in CXCR1 KO mice. Moreover, FKN potentiated glucose-induced insulin secretion (GSIS), arginine, and glucagon-like peptide-1 (GLP-1) activities.

Altogether, FKN and its interaction with CX3CR1 and integrins demonstrates a pleiotropic role for FKN over distinct physiological processes. The accumulating evidence also indicates important negative effects arising from unbalanced interactions and signaling.

## 6. Fractalkine in Cancer 

The role of FKN in cancer progression and prognosis of antineoplastic treatments is still unclear, especially considering the two main forms of FKN (Table 1). For non-small-cell lung cancer (NSCLC), there is evidence supporting a prognostic role for FKN expression both in the tumor and in plasma. Taking advantage of this observation, and the chemoattractant properties of FKN, Guo et al. transferred the full-length FKN gene to mouse 3LL lung adenocarcinoma cells, leading to a decrease in tumor growth [64]. Interestingly, genetically modified 3LL-FKN overexpressed both the membrane-bound and the soluble version of FKN. The authors demonstrated that tumors were largely infiltrated by immune cells including T cells and activated DCs. Interestingly, depletion of CD8 T cells largely abrogated the inhibition of tumor growth in this study, with a minor contribution from CD4 T cells. In a follow-up study, the authors showed that NK cells were also chemoattracted to the microenvironment of 3LL-FKN tumors. There, and possibly through interactions with activated DCs and adhesion to tumors cells, NK cells could exert cytotoxic activities towards 3LL cells that were otherwise resistant [65]. A similar approach was undertaken by expressing FKN in mouse EL-4 lymphoma cell lines, followed by transfer in wild-type, Rag1^−/−^, NK-deficient, and CXCR1^−/−^ mice [66]. Again, strong anti-tumor effects were observed in wild-type and in T-cell deficient Rag1^−/−^ mice. Tumor growth was regained in NK-deficient and CXCR1^−/−^ mice, suggesting that NK cells were indeed recruited to the tumor environment and were the main effector cytotoxic cell types. Indeed, the authors demonstrated that IFNγ and perforin were required for FKN-dependent anti-tumor activities. These data were supported by a recent study in which a major role for NK cells was demonstrated for FKN-mediated anti-tumor immunity in NSCLC, at least for soluble FKN [67]. 

High tumor infiltration by lymphocytes (tumor-infiltrating lymphocytes, or TILs) is a well-known and good prognostic marker in colorectal cancer. Indeed, a positive correlation was found between increased FKN expression quantified via immunohistology and high TIL infiltration including NK cells, in biopsies from colorectal cancer patients [68]. Hence, the authors of this study assigned a good prognostic value for elevated FKN expression in colorectal tumors. Overall, similar associations of FKN with good prognosis for cancer treatments have been demonstrated for other cancer types, such as in neuroblastoma [69]. Thus, NXS2 cells were genetically modified to overexpress the full-length FKN transgene. Interestingly, supernatants from these modified cells demonstrated effective chemoattracting properties for leukocytes, suggesting the secretion of sFKN. NXS2-FKN cells showed decreased cell growth but also diminished capacities to produce spontaneous liver metastases. Nevertheless, it is important to point out that this study used subcutaneous cancer cell transplantation, rather than intracranial transplantation. Importantly, the anti-tumor effects achieved by FKN gene transfer were potentiated by combination with IL-2-based immunotherapy. 

The anti-tumor properties of FKN were utilized in a cancer vaccination model using dendritic cells genetically modified with an adenovirus encoding the full-length fractalkine gene [70]. Interestingly, supernatants from FKN-DC showed enhanced chemotactic properties in vitro, suggesting that soluble FKN was also produced by modified DCs. Significant therapeutic activities were demonstrated in two cancer models, murine B16F10 melanoma and a mouse colorectal cancer model based on colon-26 cells. Enhanced anti-tumor cytotoxic T lymphocyte (CTL) responses were induced by FKN-DC, and in these two models, therapeutic activities were completely abrogated in mice lacking either CD4 or CD8 T cells. These results suggested that T-cell responses were required for the anti-tumor activities of DCs expressing FKN. In a study of the expression of FKN and its receptor in gastric adenocarcinoma analyzed with immunohistochemistry and western blot in hepatocellular carcinoma samples, the authors showed better prognosis and longer survival when both FKN and its receptor were expressed [71]. Anti-tumor capacities of the FKN full-length gene were also demonstrated in mouse models of hepatocellular carcinoma as well. This was demonstrated via genetic modification of mouse hepatocellular carcinoma MM45T.Li cells and transfer into immunocompetent mice. This approach achieved significant inhibition of tumor cell growth, which correlated with CD4 and CD8 T-cell infiltration within the tumors and the induction of systemic CTL responses [72]. In agreement with this study, Vitale and co-workers performed a detailed analysis of the anti-tumor activities of three molecular forms of FKN in the mouse C26 colorectal cancer model [73]. These three forms included the native FKN full-length gene, a membrane-bound FKN, and a soluble FKN form. C26 cells were genetically modified to express these FKN variants following subcutaneous transplantation into mice. The capacities of FKN for chemoattraction of NK cells were shown. Interestingly, the three forms exhibited anti-tumor activities in this model, but also for liver tumors and lung metastases. In this latter case, the membrane-bound FKN did not show any benefit. Moreover, the authors showed some differential effects depending on the location of the tumor, and the relative contribution of CD4 and CD8 T cells to tumor rejection in depletion experiments. As FKN had a positive effect over anti-tumor activities in colorectal cancer, Siddiqui et al. modified human T cells to express the FKN receptor, which favored trafficking to FKN-expressing tumors with a resulting decrease in tumor growth through CTL activities in adoptive transfer experiments in mice [74]. Neuroblastoma is a type of cancer in which FKN is abundantly expressed. In a mouse model of neuroblastoma, FKN expression alone was not sufficient to elicit anti-tumor activities [75]. However, combination with an IL-2-based immunotherapy achieved significant therapeutic activities with complete eradication of liver metastases. In this case, T-cell and NK cell depletion abrogated therapeutic activities. A study of breast carcinoma patients demonstrated that those with elevated FKN expression correlated with a better prognosis and prolonged overall survival, compared to patients with reduced FKN within tumors [76]. This better prognosis was associated with an enhancement in the recruitment of immune cells in the stroma, including CD8 T cells, NK cells, and DCs. A similar result was observed in patients with soft tissue sarcomas, in which increased FKN expression was associated with reduced BAX expression which correlated with better prognosis [77]. Interestingly, in this study, the authors showed that FKN expression was associated with reduced proliferation of cancer cells, possibly through an inhibition of apoptosis when specifically silencing FKN. These direct anti-tumor effects over cancer cell proliferation and apoptosis were also demonstrated in NSCLC cells [67]. Nevertheless, the correlation between FKN expression as assessed via different methods and for different cancer types and prognosis and survival of patients may differ. Su and co-workers found that the median survival time of NSCLC patients with a history of smoking was reduced when FKN expression was increased in the tumor, as assessed via immunohistochemistry [78]. No such adverse effects were observed in lung adenocarcinoma patients without history of smoking, or in squamous cell carcinoma with a history of smoking. The authors of the study provided evidence that FKN enhanced cell invasion of tumor cells by inducing MMP2/MMP9 expression through the activity of JNK. However, several studies show the opposite result through, for example, evaluating FKN mRNA expression levels in tumors [79] or quantifying soluble FKN in plasma in NSCLC patients [67]. In these two last studies, elevated FKN expression was associated with improved prognosis and better response to therapies through the anti-tumor activities of T cells and NK cells. 

In some circumstances, FKN expression can be regulated by cancer cells as a consequence of the accumulation of mutations. Interestingly, the presence of the R132H mutation in IDH1 mutations is associated with better prognosis in glioma patients. Ren and collaborators showed that IDH1-R132H was inducing the expression of FKN by cancer cells, which in turn favored the recruitment of NK cells with cytotoxic activities [80]. In this study FKN expression was studied via quantification of mRNA levels, Western blot, and assessing its chemoattractant potential. Elevated FKN expression was also demonstrated to be a good prognostic feature in breast cancer [81]. When overexpressed in several breast cancer cell lines, a recruitment of T cells and NK cells was achieved, which mediated tumor cell killing and showed synergistic effects with trastuzumab treatment. Indeed, FKN overexpression overcame trastuzumab resistance. Similar therapeutic results were confirmed by Zhang and co-workers who found that macrophages contributed to the resistance of tumor cells to IL-15 through a regulatory axis that involves regulation of FKN expression [82]. IL-15Rα(+) TAMs reduced the FKN expression in tumor cells, which caused the inhibition of the recruitment of CD8 T cells. The authors also demonstrated that HIF-1α ultimately regulated FKN expression in tumor cells in this experimental system. Interestingly, preinfusion levels of IL15, Flt3-L, and FKN in clinical products of transgenic T cell receptor (TCR) T cells targeting MART-1 and NYESO-1 in clinical trials for melanoma correlated with response to treatment [83]. These results suggest that incorporation of these cytokines including FKN into T cell-based therapy products could have a beneficial effect at least for the treatment of melanoma. 

Nevertheless, there are some reports that link increased FKN expression or its receptor in tumors with worse prognosis. Some of these are in contrast to other studies that associate the FKN–CX3CR1 signaling axis with good prognosis. For example, a correlation study using the human protein atlas data showed worse prognosis with increased FKN expression in some cancer types, although statistical significance was reached only for stomach, liver, and urothelial cancer [41]. Conversely, increased CX3CR1 expression was found to be associated to worse prognosis in a number of cancer types, reaching statistical significance for testis cancer. Nevertheless, it could be argued that since FKN and CX3CR1 expression was evaluated mainly with mRNA expression [84], no information was available on protein expression levels, or the specific FKN forms that are expressed. Other studies link the upregulation of the FKN/CX3CR1 axis in tumorigenesis with enhanced metastasis and invasion, although in some cases it simultaneously promotes tumor infiltration with effector immune cells. These seemingly contradictory results, or at least paradoxical situations, have been shown for breast cancer [46,85,86], prostate cancer [87,88,89,90], melanoma [91], multiple myeloma [92,93], gastric cancer [94,95], pancreatic cancer [96,97,98], ovarian carcinoma [99], leukaemia [100], and clear-cell renal carcinoma [44]. Although the FKN–CX3CR1 axis is overall associated with good prognosis in lung cancer, Liu and collaborators showed that FKN expression promotes cell invasion and metastasis at least in a large-cell lung cancer cell line [47]. 

**Table 1:** Fractalkine in cancer.

FKN Function	Cancer Type and Model	Mechanisms	References
Anti-tumor	Murine lung cancer 3LL cells	CD8 and NK cells	[64,65]
Anti-tumor	Murine EL4 lymphoma cells	NK cells	[66]
Anti-tumor and good prognosis	NSCLC patients and murine lung cancer cells	NK cells, reduced proliferation of cancer cells	[67]
Good prognosis	Human colorectal cancer	TIL infiltration	[68]
Anti-tumor	Murine NXS2 neuroblastoma	Combination with IL-2 therapy	[69]
Anti-tumor	Murine B16F10 melanoma and colon-26 cancer models with DC-FKN transfer	CD4 and CD8 T cells	[70]
Good prognosis	Human gastric adenocarcinoma and hepatocellular carcinoma; FKN expression	Correlation with expression	[71]
Anti-tumor	Murine hepatocellular carcinoma MM45T.Li cells	CD4 and CD8 T cells	[72]
Anti-tumor	Murine C26 colorectal cancer model	CD4 and CD8 T cells	[73]
Anti-tumor	Human colorectal cancer cells in mouse xenograft	CTL activities	[74]
Good prognosis	Human breast carcinoma	Elevated expression, CD8, DC, and NK infiltration	[76]
Good prognosis	Human soft tissue sarcomas	Elevated expression, reduced proliferation of cancer cells	[77]
Worse prognosis	NSCLC patients with a history of smoking	Enhanced cancer cell invasion	[78]
Good prognosis	NSCLC patients	Elevated mRNA expression in tumors	[79]
Good prognosis	Glioma patients	NK recruitment and activity	[80]
Good prognosis	Breast cancer	T and NK cell recruitment, synergy with trastuzumab	[81]
Anti-tumor	Mouse breast cancer	CD8 T cells	[82]
Anti-tumor	Human melanoma	As a therapy associated with TCR-modified T cell transfer	[83]
Worse prognosis	Stomach, liver, and urothelial cancer	Correlation studies	[41]
Worse prognosis	Testis cancer and prostate cancer	Elevated FKN–CX3CR1 signaling axis	[84,87,88,89,90]
Worse prognosis	Breast cancer	Elevated FKN–CX3CR1 signaling axis	[46,85,86]
Pro-tumor effects	Murine B16 melanoma	Silencing of surface FKN delays tumor growth	[91]
Worse prognosis	Human multiple myeloma patients and cell lines	Elevated expression in bone marrow and in tumor cells	[92,93]
Worse prognosis	Human gastric cancer samples and cell lines	Elevation of CX3CR1 and FKN in cancer cells	[94,95]
Pro-tumor	Human pancreatic cancer	Resistance to apoptosis, reprogramming of glucose metabolism	[96,97,98]
Pro-tumor and worse prognosis	Human ovarian carcinoma	Enhanced proliferation of cancer cells through AKT activation	[99]
Pro-tumor	Human leukemia	Invasion of cancer cells	[100]
Worse prognosis	Human clear-cell renal carcinoma	Increased CX3CR1 expression	[44]

## 7. Fractalkine in Cancer Immunotherapy

### 7.1. Immune Checkpoint Blockade Immunotherapies

**Figure 3:**
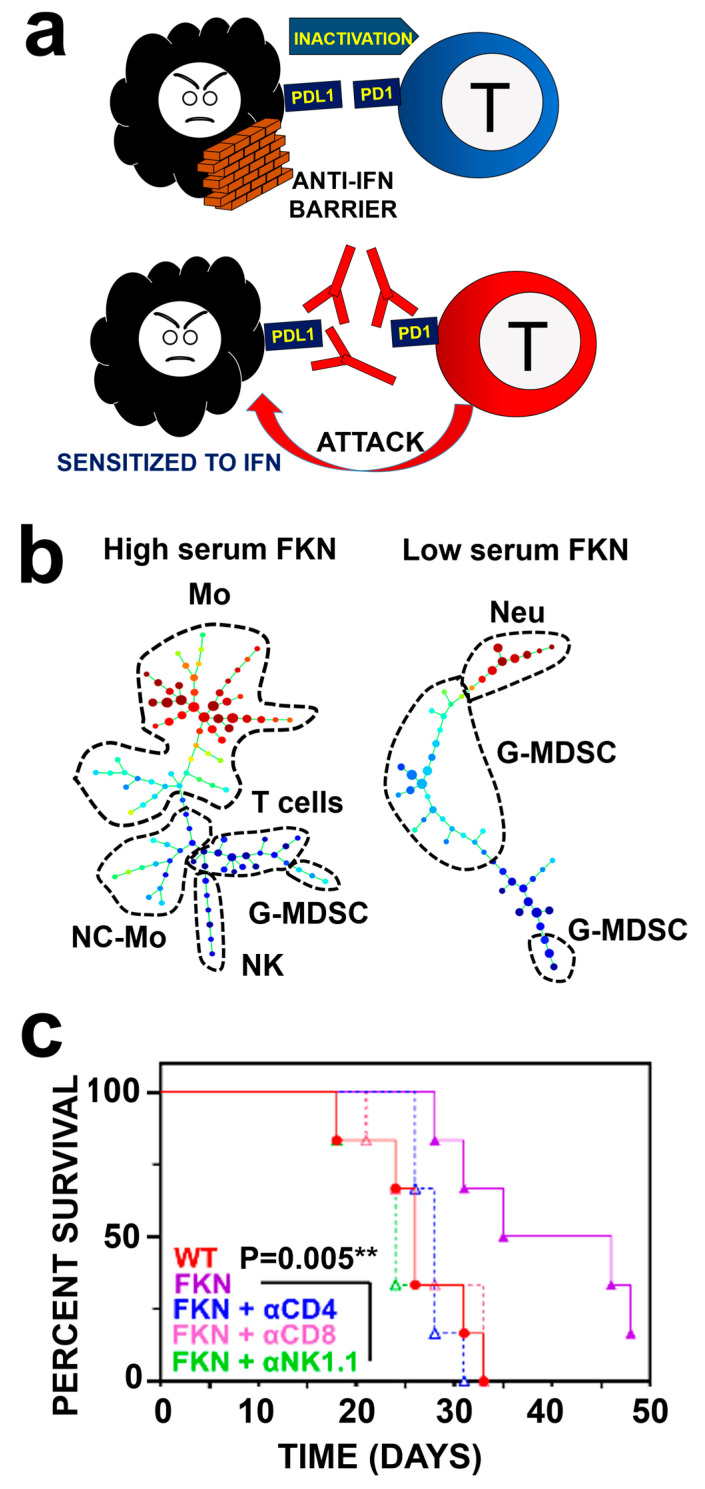
FKN in cancer immunotherapy. (**a**) **Top**: cancer cells (black) express PD-L1 on their surface that binds PD-L1 on the surface to T cells (blue cell on the left). This interaction inhibits T cell effector activities and creates a signaling barrier that protects cancer cells from interferon-induced apoptosis. **Bottom**: anti-PD-1 and anti-PD-L1 antibodies block PD-1/PD-L1 interactions, leading to enhanced T cell cytotoxicity towards cancer cells and increased sensitivity to interferon-induced apoptosis in cancer cells. (**b**) Plasma soluble FKN is a biomarker for immune cell diversity in peripheral blood from human NSCLC patients. **Left**: SPADE 3 hierarchical clustering of immune cell types in peripheral blood identified via high-dimensional flow cytometry in long-term responder patients to PD-1/PD-L1 blockade. **Right**: same as left but in non-responder patients [67]. The number of branches indicate the phenotypic diversity of immune cell types. Major lineages are grouped and indicated in the cluster trees. Mo, monocytes; NC-Mo, non-classical monocytes; G-MDSCs, granulocytic myeloid-derived suppressor cells; Neu, neutrophils. NK, natural killer cells. (**c**) FKN anti-tumor activities are dependent on NK and T cells. The graph shows a Kaplan–Meier survival plot in mice transplanted with lung cancer cells expressing soluble FKN, in which CD4, CD8, or NK cells have been depleted. FKN-dependent anti-tumor activities are eliminated following NK and T cell depletion [67]. **, indicates very significant differences by the Log-Rank test (*p* < 0.01).

The development of clinically successful immunotherapies has revolutionized oncology in the last decade [101]. The discovery of immune checkpoint molecules was central to the development of these strategies. Immune checkpoint molecules are key regulators of immune responses, and their physiological functions are related to maintaining systemic tolerance towards auto-antigens or to limit autoreactive damage in infection and inflammation [102,103,104,105]. The first immune checkpoint molecule to be clinically targeted with blocking antibodies was CTLA-4 [106]. Nevertheless, the most successful immune checkpoint inhibitors in clinical oncology are the two partners of the PD-1/PD-L1 signaling pathway [104,107,108] (Figure 3a). PD-1 is transiently expressed on the surface of activated T cells and constitutively in exhausted and anergic T cells. PD-L1 is the ligand of PD-1, and it is expressed by a wide variety of cell types, including antigen-presenting cells of the myeloid lineage such as dendritic cells, neutrophils, and myeloid-derived suppressor cells, as well as by tumor cells [105]. When PD-1 is engaged with PD-L1, it transmits inhibitory signals to T cells through several mechanisms, leading to cell cycle arrest and termination of T-cell effector activities [104]. This interaction frequently takes place within the tumor environment, although it can also take place systemically within secondary lymphoid organs during physiological antigen presentation. PD-L1 provides additional back-signaling to the cancer cell which enhances resistance to pro-apoptotic insults, such as interferon and other cytotoxic molecules [109].

PD-1/PD-L1 blockade immunotherapy consists of systemic administration of antibodies that block this interaction with the aim of reactivating T-cell responses towards cancer cells. This immunotherapy strategy is without any doubt the most successful to-date, demonstrating therapeutic activities in many cancer types. PD-1/PD-L1 blockade immunotherapy is administered as a first line treatment alone or in combination with chemotherapy is some cancers, such as in non-small-cell lung cancer (NSCLC) [110].

### 7.2. Fractalkine as a Biomarker of Response in Lung Cancer Immunotherapy

While immune checkpoint blockade has yielded remarkable clinical results, a significant proportion of cancer patients fail to respond to these therapies [111]. For example, NSCLC remains a leading cause of death in which PD-1/PD-L1 blockade therapies fail in more than 50% of treated patients. Therefore, there is an urgent need to find biomarkers of response to these therapies that could help stratifying patients by selecting those with a high probability of benefiting from therapy. Moreover, the identification of biomarkers of response could shed light into strategies to overcome resistance to treatment [112].

There is increasing evidence demonstrating that functional systemic immunity is required even before starting immunotherapies to achieve good immunotherapy outcomes [110,112,113,114,115,116,117]. For example, our group and others have shown that functional systemic CD4 T-cell immunity is a key requirement [110,112,113,114,115]. T cell responses are ultimately dependent on antigen presentation by antigen-presenting cells (APCs) from the myeloid lineage [118,119]. Hence, elevated proportions of HLA-DR^+^ monocytes and decreased neutrophils in peripheral blood correlates with clinical responses to immunotherapies [119,120,121]. In a recent study, we carried out a high-throughput myeloid cell profiling in peripheral blood to identify cell populations associated with response to PD-1/PD-L1 blockade in NSCLC (Figure 3b). Interestingly, an elevated diversity of myeloid cell types was a biomarker for objective responses [67], possibly reflecting the need for functional myelopoiesis in patients [122]. These results were in agreement with previous published studies using high-dimensional analytical techniques [120,123,124]. 

Interestingly, elevated concentrations of soluble FKN in plasma before starting immunotherapies was associated with high systemic myeloid cell diversity, increased monocyte populations, and prolonged survival in NSCLC patients [67]. Indeed, FKN served as a biomarker with potential predictive power at least in NSCLC for PD-1/PD-L1 blockade immunotherapies. In agreement with these observations, it is known that, overall, increased FKN expression within the tumor environment largely correlates with good prognosis, although not in all cancer types [41,68]. The relationship between myeloid cell diversity and plasma sFKN was also confirmed in mouse models of lung cancer. Hence, elevation of sFKN plasma levels by FKN-overexpressing implanted tumors in mice correlated with increased numbers of monocytes and a reduction in Ly6G+ granulocytes in the spleen and peripheral blood.

In a recent study by Cappelletto et al., the authors evaluated a large collection of cytokines with biomarker potential which included FKN together with soluble PD-L1 in plasma and sera from melanoma and NSCLC patients treated with anti-PD-1 and anti-PD-L1 immunotherapies [125]. In this study, the authors set up a Luminex assay to simultaneously quantify the cytokines. FKN could be detected at good levels, although the authors did not perform any study of the correlation to response due to the limited number of patients.

### 7.3. FKN as a Therapeutic Anti-Cancer Agent

The overexpression of sFKN by cancer cells has been demonstrated to have anti-cancer properties. Thus, sFKN exerts anti-proliferative effects in cultures of cell lines, for example, when directly expressed by cancer cells [64,67]. Transplantation of cancer cells engineered to secrete FKN in mouse models for lung adenocarcinoma leads to a significant reduction in tumor growth [64,67], which can be replicated by systemic administration of recombinant protein [67]. Moreover, pharmacological disruption of signaling by the FKN receptor using a small molecule inhibitor accelerates tumor growth in mouse models. Nevertheless, these anti-cancer effects were not caused only by inhibition of cancer cell proliferation, but also through the induction of anti-tumor immune responses leading to tumor rejection [67]. This was also the case for FKN expression in hepatocellular carcinoma cells, which were rejected following transplantation into immunodominant mice through immune-mediated mechanisms [72]. Indeed, secreted sFKN was shown to inhibit metastases in neuroblastoma mouse models through a mechanism dependent on T cells and NK cells [75]. Hence, most of the experimental evidence points to distal anti-tumor effects through sFKN. For example, sFKN produced by one tumor exerted inhibitory effects over FKN-non-expressing distal tumors in a mouse model for lung adenocarcinoma [67]. Therefore, several mechanisms must take place when using sFKN as a therapeutic agent, apart from the well-known recruitment of effector T cells, NK cells, or DCs towards the tumor environment [35,36,66,68,72,76,126,127,128]. 

The anti-tumor properties of sFKN have been ascribed to several immune cell types, which include T cells, myeloid cells such as dendritic cells, and NK cells [64,66,67,70,72,74,75,126]. In fact, in vivo NK depletion was shown to abrogate the anti-tumor properties of sFKN, and CD4 and CD8 T-cell depletion also contributed to therapeutic efficacy in a mouse model of lung cancer, although these results were obtained in combination with anti-PD-1 immunotherapy [67] (Figure 3c). Indeed, expression of sFKN in mouse lung adenocarcinoma cancer models refractory to anti-PD-1 therapies [129] sensitized these tumors to PD-1 blockade, delaying tumor growth. Current evidence suggests that these effects are largely NK cell-dependent, at least in lung adenocarcinoma [65,67]. It is important to remark that anti-tumor properties have been described for sFKN, while, in some cancers, it has been shown that membrane-bound FKN promotes metastasis of CX3CR1^+^ cancer cells towards sites with elevated CX3CL1 expression, such as lungs, bones, and others [41,45,92,99,130,131,132,133]. These results strongly support a dual role for FKN associated with the two main bioactive forms [73,134,135]. The membrane FKN promotes cell adhesion and migration, which could also favor metastasis of cancer cells. For example, in colon carcinoma CT26 engineered to express CX3CR1, the use of an FKN–CX3CR1 blocking antibody in combination with anti-PD-1 results in enhanced therapeutic activities [37]. On the other hand, sFKN contributes to chemoattraction of immune cells to the tumor, favoring anti-tumor activities when in combination with immunotherapy [64,65,67,74]. Recently, deficiencies in the FKN-receptor signaling axis were found to be responsible for the exclusion of immune cells from the tumor microenvironment [136]. To overcome this barrier, NKG2D-CAR-T cells were engineered to express the FKN receptor to improve their infiltration within tumors, and improved efficacy of CAR-T therapies were demonstrated. 

## 8. Concluding Remarks

The function of the FKN–CX3CR1 axis is regulated by different mechanisms. As any other gene, it is transcriptionally regulated. However, the translational product of the fractalkine gene is also under the control of different mechanisms. Once generated, the protein can be presented on the cell surface or recycled into endosomes, where it is retained until it is needed. Moreover, fractalkine is a substrate for different proteases that, through its shedding, promotes acquisition of chemotactic activities or the elimination of its biological activities. Finally, biologically active soluble protein binds integrins causing their activation followed by their binding to their respective numerous ligands. This is a highly complex regulatory mechanism, as we have to take into account that each integrin can bind to various ligands promoting different cell activities, depending on the context. While inside-out integrin signaling requires fractalkine signaling through its receptor, outside-in signaling takes place in the absence of CX3CR1. All these accumulating data indicate that the role of fractalkine in cancer or other pathological conditions cannot be delimited to interactions with its cognate receptor. To explain the pro- or anti-tumor immunity of fractalkine we cannot just focus on its interaction with its receptor alone. The role of fractalkine in different integrin activations is an extremely important element that has to be considered in tumor progression and/or anti-tumor response establishment as another element of this complex puzzle picture. 

So far, FKN has been associated with numerous malignancies (www.malacards.org; 678 diseases matching CX3CL1; date of accession 18 July 2024). Therefore, understanding the exact mechanisms of action in different disease contexts (oncological malignancies, autoimmune disorders, and neurodegenerative diseases, for example) is valuable. The published studies and gathered data generated so far have allowed for the realization of different clinical trials (www.clinicaltrials.gov; search terms “fractalkine”; date of accession 18 July 2024). 

In the numerous clinical trials, FKN is used as a potential biomarker in different neoplastic (NCT02774395, endometrial cancer; NCT04576429, melanoma; and NCT04253145, small-cell lung cancer), autoimmune (NCT05387473, osteoarthritis and NCT04995588, systemic sclerosis), neurodegenerative, and cognitive disorders (NCT04506073, Parkinson’s disease; NCT06337539), as well as infectious diseases (NCT06444893, COVID19 and NCT04870138, gonococcal infection). Quantification of FKN as a biomarker is frequently performed in combination with other biomarkers of disease or of response, utilizing techniques such as ELISA or Luminex. One of these clinical trials currently in the recruitment stage utilizes FKN as an anti-cancer drug in combination with chemotherapy (NCT06087289). 

## Data Availability

Not applicable.
